# Combined Effects of Methylated Cytosine and Molecular Crowding on the Thermodynamic Stability of DNA Duplexes

**DOI:** 10.3390/ijms22020947

**Published:** 2021-01-19

**Authors:** Mitsuki Tsuruta, Yui Sugitani, Naoki Sugimoto, Daisuke Miyoshi

**Affiliations:** 1 Faculty of Frontiers of Innovative Research in Science and Technology (FIRST), Konan University, Kobe 650-0047, Japan; m2061010@s.konan-u.ac.jp (M.T.); sugitani@jcdb.co.jp (Y.S.); sugimoto@konan-u.ac.jp (N.S.); 2 Frontier Institute for Biomolecular Engineering Research (FIBER), Konan University, Kobe 650-0047, Japan

**Keywords:** methylated cytosine, molecular crowding, osmolyte, epigenetics, thermodynamics

## Abstract

Methylated cytosine within CpG dinucleotides is a key factor for epigenetic gene regulation. It has been revealed that methylated cytosine decreases DNA backbone flexibility and increases the thermal stability of DNA. Although the molecular environment is an important factor for the structure, thermodynamics, and function of biomolecules, there are few reports on the effects of methylated cytosine under a cell-mimicking molecular environment. Here, we systematically investigated the effects of methylated cytosine on the thermodynamics of DNA duplexes under molecular crowding conditions, which is a critical difference between the molecular environment in cells and test tubes. Thermodynamic parameters quantitatively demonstrated that the methylation effect and molecular crowding effect on DNA duplexes are independent and additive, in which the degree of the stabilization is the sum of the methylation effect and molecular crowding effect. Furthermore, the effects of methylation and molecular crowding correlate with the hydration states of DNA duplexes. The stabilization effect of methylation was due to the favorable enthalpic contribution, suggesting that direct interactions of the methyl group with adjacent bases and adjacent methyl groups play a role in determining the flexibility and thermodynamics of DNA duplexes. These results are useful to predict the properties of DNA duplexes with methylation in cell-mimicking conditions.

## 1. Introduction

The covalent addition of a methyl group to the 5-position of the cytosine ring within a CpG dinucleotide is an epigenetic modification of DNA that is vital for cellular development [1,2,3]. In vertebrate genomes, approximately 70% of CpG residues are methylated [4]. CpG islands are relatively short (~1 kb) sequences that frequently coincide with gene promoter regions, especially nearby transcription start sites [5,6]. Moreover, next-generation sequencing of the methylome for the whole genome has identified methylation in non-CpG contexts (mCH, where H=A, C, or T) in stem cells, oocytes, and neurons of mammals [7,8,9,10,11,12,13]. mCH is largely depleted from embryo stem cells upon cell differentiation and is restored in induced pluripotent stem cells [14]. Proper DNA methylation is a prerequisite for normal development and is further involved in various biological processes such as the regulation of gene expression, X-chromosome inactivation, suppression of repetitive genomic elements, and carcinogenesis [15,16,17,18]. Moreover, deregulation of epigenetic gene expression is related to the onset of cancer and neurodegeneration [19,20]. It is generally considered that methylated cytosine represses gene expression by binding with methylation binding proteins, resulting in the blocking of transcription factors [21,22,23]. Thus, the specific binding of methylation binding proteins to methylated DNA is critical for the epigenetic gene regulation system. The affinity between methylated DNA and methylation binding proteins is affected by the DNA structure. Therefore, the effects of methylated cytosine on the DNA structure and its stability and dynamics in living cells are important not only for understanding epigenetic mechanisms, but also for developing tailored epigenetic therapies that target cancer and neurodegeneration.

Structural studies with NMR and X-ray crystallography have demonstrated that methylated cytosine reduces the dynamics of the backbone of DNA duplexes [24,25,26,27,28,29]. In agreement with these experimental studies, molecular dynamics (MD) studies have also indicated that DNA flexibility is reduced by steric repulsion and hydrophobicity of the methyl groups [30,31]. Moreover, the effects of methylated cytosine on the thermal stability of DNA structures have also been reported. Nardo and Mantegazza systematically conducted FRET melting analysis on DNA duplexes with various numbers of methylated cytosines under a wide cation concentration range [29]. They found that the melting temperature values were systematically upshifted by approximately 0.5 °C per methylated cytosine. This thermal stabilization is consistent with a study by Marky and colleagues using differential scanning calorimetry [32]. Methylation effects on the thermal stability of noncanonical DNA structures, triplexes, and i-motifs have also been reported [26,32,33,34]. However, most of these biophysical and biochemical studies on methylation effects have been carried out in test tubes with dilute buffer conditions that are totally different from those in living cells, where DNA folds into various structures to play critical roles in various biological processes.

In addition to methylated cytosine, the cellular environment has significant effects on the structure and thermodynamic stability of DNA. Cellular environmental factors such as pH, cations, and temperature influence DNA thermodynamic stability [35]. Moreover, the volume of the cell is constituted by 20–40% biomolecules, leading to molecular crowding [35]. It has been demonstrated that B-form duplexes via Watson–Crick base-pairing are destabilized under molecular crowding conditions, whereas G-quadruplexes and triplexes with Hoogsteen base pairs are stabilized [36,37,38]. From these results, it is possible to consider that molecular crowding, which is a major difference between cell and test tube conditions, is a critical factor in determining the structure and stability of DNA. Currently, there have been only a few reports on the methylation effect on the thermal stability of DNA under molecular crowding conditions. Shao’s group reported the methylation effects on thermal stability of human telomeric i-motif structures under molecular crowding conditions with Ficoll 400 [39]. They found that the methylation effects on thermal stability of the i-motif were conserved under crowding conditions. Conservation of the cytosine effect under molecular crowding conditions has also been observed for DNA triplexes [32]. In contrast, Wadkins and colleagues studied the methylation effects on *c-myc* i-motif DNA and found that the effects on stability were abolished under molecular crowding conditions with small molecules having different numbers of hydroxyl groups [37]. Although the discrepancy in these studies could be due to differences in DNA sequences and cosolutes in the experiments, further studies are required to unravel the properties of epigenetically modified DNA oligonucleotides under cell-mimicking crowded conditions. To understand the effects of methylation under crowded conditions on the thermodynamics of DNA, the duplex structure is suitable for the following reasons. First, oligonucleotide duplexes are appropriate for thermodynamic analysis based on a two-state transition model, whereas i-motif and triplex structures often do not follow a two-state transition. Second, the duplex may maintain its structure in spite of various nucleotide modifications, which makes it possible to alter the nucleotide sequence in a systematic manner. Third, the DNA duplex is not sensitive to cation species and solution pH, simplifying quantification of the methylation effects.

Here, we systematically examined the thermodynamic properties of methylated DNA oligonucleotides under molecular crowding conditions with various cosolutes such as polyethylene glycol (average molecular weight 200 (PEG200)), trimethylamine *N*-oxide (TMAO), urea, and L-proline to elucidate the effects of DNA methylation in a cell-mimicking environment. Thermodynamic parameters as well as CD spectra demonstrated that the effects of methylation and molecular crowding are independent and additive. Thermodynamic parameters had a linear relationship with the number of methylations and the degree of molecular crowding. Moreover, we found that the stabilization effect of methylation was due to the favorable enthalpic contribution, suggesting that direct interactions of the methyl group with adjacent bases and adjacent methyl groups play a role in determining the flexibility and thermodynamics of DNA duplexes. These results are useful to predict the properties of DNA duplexes with methylation in cell-mimicking conditions.

## 2. Results

### 2.1. Design of DNA Sequences and Crowding Reagents

**Figure 1:**
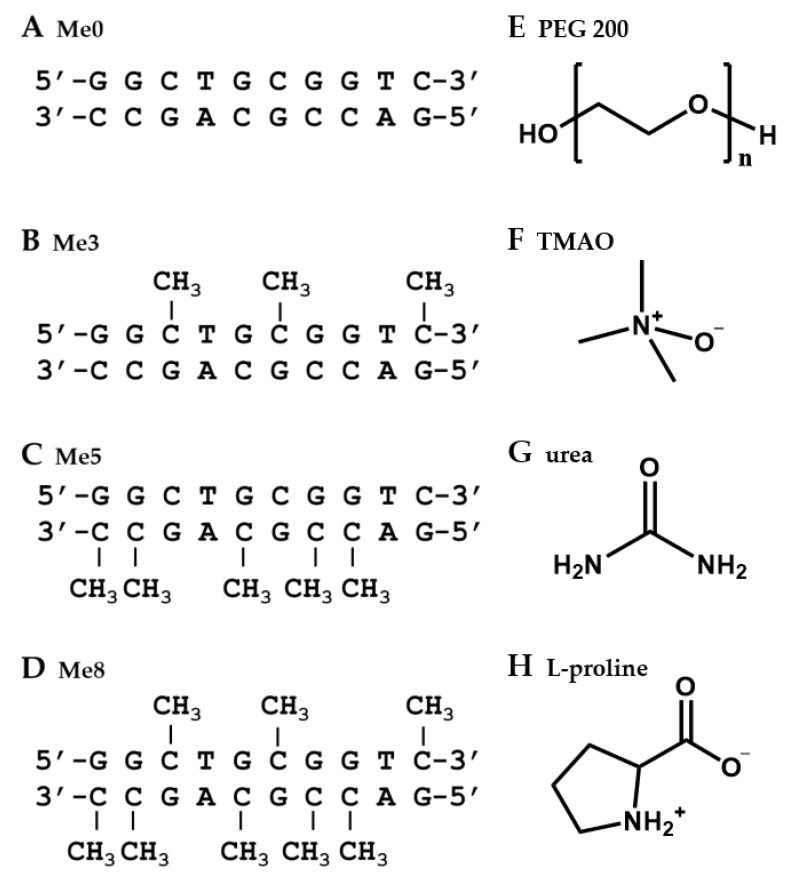
Sequences of DNA oligonucleotides and chemical structure of cosolutes used in this study. (**A**) Me0. (**B**) Me3. (**C**) Me5. (**D**) Me8. Chemical structures of (**E**) PEG200, (**F**) TMAO, (**G**) urea, (**H**) L-proline.

We designed 10-mer non-self-complementary DNA oligonucleotides (Figure 1A–D). In order to systematically investigate the methylated cytosine effects, the DNA oligonucleotides comprised various numbers of methylated cytosines (Me0: without methylation, Me3: with three methylations, Me5: with five methylations, and Me8: with eight methylations). These oligonucleotides were designed to show a two-state transition in the formation of the duplex with a moderate thermal stability to evaluate the methylation and molecular crowding effects.

As cosolutes to induce molecular crowding, PEG200 (Figure 1F) was used as a standard cosolute as described previously [40,41,42,43]. We have used PEG200 to evaluate molecular crowding effects on structural stability of various nucleic acids interpreted the results from our and other groups as a review paper [40,41,42,43]. Therefore, PEG200 is suitable cosolute to compare with previous studies for molecular crowding effects. PEGs have been used as a cosolute because of the following reasons. First, PEGs are basically inert with biomolecules. Second, PEGs are highly water soluble. Although none PEGs and the naturally occurring cosolutes do not completely mimic intracellular conditions, it is important to use such inert and water soluble cosolutes to evaluate biomolecular characters such as thermodynamics and hydration under molecular crowding conditions. In addition to the above-mentioned reasons, PEGs with various molecular weights are commercially available and are inexpensive to use in great quantities as cosolutes. Notably, it was recently reported that nucleic acid structure in a molecular crowding condition with PEG200 is similar with that in cell nucleolus, where DNA molecules exist [44].

TMAO, urea, and L-proline were also used as low molecular weight cosolutes. TMAO is zwitterionic and a protective osmolyte for various proteins [45,46,47]. We also reported that TMAO stabilizes DNA duplexes due to its preferential interactions with nucleotide bases [38]. Urea is an important cosolute with implications in maintaining osmotic stress in cells and destabilizing biomolecular structures [47,48,49]. L-proline is a compatible cosolute that accumulates in plants, bacteria, algae, and marine invertebrates in response to osmotic stress [50,51]. L-proline stabilizes proteins because of its unfavorable interactions with various functional groups on the surfaces of proteins, resulting in preferential interactions with water molecules [52]. Since the total concentration of biomolecules in a living cell is around 20–40 wt%, here we added cosolutes up to 40 wt% to induce model molecular crowding conditions.

### 2.2. Effects of Methylation and Molecular Crowding on DNA Structure

**Figure 2:**
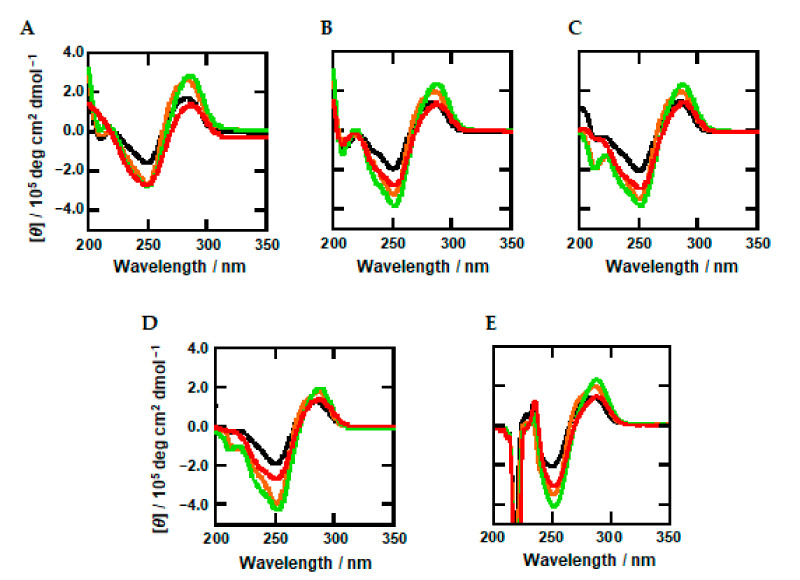
CD spectra of 20 μM oligonucleotides. CD spectra in a buffer containing 100 mM KCl, 10 mM K_2_HPO_4_ (pH 7.0), and 1 mM K_2_EDTA in the absence of cosolutes (**A**) or in the presence of 30 wt% cosolutes (PEG200 (**B**), TMAO (**C**), urea (**D**), and L-proline (**E**)) at 25 °C. Me0: black, Me3: orange, Me5: green, and Me8: red.

First, we studied the structures of the DNA oligonucleotides using circular dichroism (CD) spectroscopy. Figure 2A shows the CD spectra of 20 μM oligonucleotides (Me0, Me2, Me3, Me5, and Me8) in a buffer containing 100 mM KCl, 10 mM K_2_HPO_4_ (pH 7.0), and 1 mM K_2_EDTA at 25 °C. Although peak position and intensity depend on the oligonucleotide sequence, these CD spectra showed positive and negative peaks around 280 nm and 250 nm, respectively, indicating the B-form duplex for all oligonucleotides [53]. In addition, it is possible that minor changes in the CD spectra of the DNA oligonucleotides reflect dynamics and flexibility of the DNA backbone as will be discussed later.

CD spectra of the oligonucleotides in the presence of 30% PEG200, TMAO, urea, and L-proline are shown in Figure 1B–E, respectively. These CD spectra were similar to that in the absence of cosolutes, showing that the B-form duplex is maintained under molecular crowding conditions with cosolutes. In addition, a sharp negative peak around 220 nm was observed in the presence of L-proline (Figure 2E). This signal is not from the oligonucleotide but from highly concentrated L-proline, which has a CD intensity around 220 nm. These results show that the oligonucleotides maintain the B-form duplex under molecular crowding conditions, although methylation may affect the flexibility of DNA strands and induce local structure changes.

### 2.3. Thermal Stability of DNA Duplexes with Methylations under Dilute and Molecular Crowding Conditions

**Figure 3:**
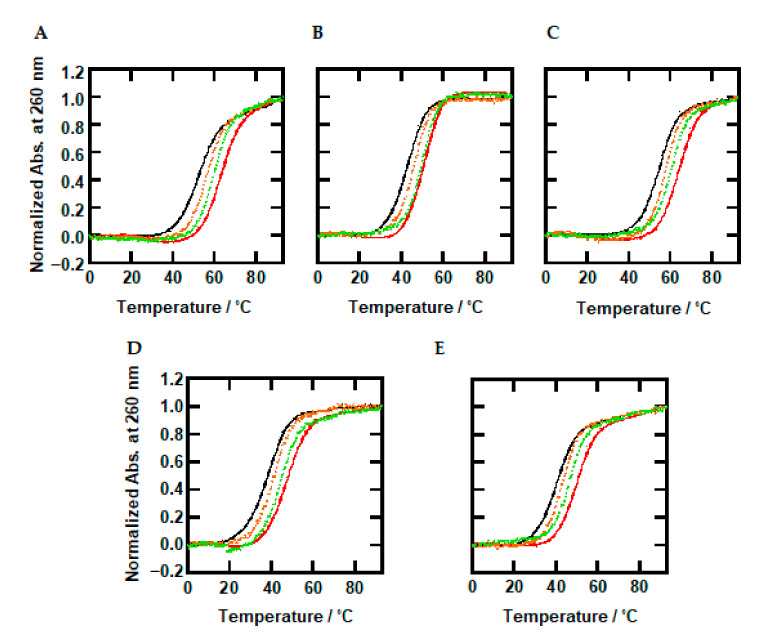
Normalized UV-melting curves for 2.0 µM Me0, Me3, Me5, and Me8. Normalized UV-melting curves in the 100 mM KCl buffer in the absence of cosolutes (**A**) or in the presence of 30 wt% PEG200 (**B**), TMAO (**C**), urea (**D**), or L-proline (**E**). UV melting curves were traced at 260 nm. Me0: black, Me3: orange, Me5: green, and Me8: red.

To investigate the combined effects of molecular crowding and methylated cytosine on the thermodynamics of the B-form DNA oligonucleotides, we performed UV-melting analysis in the absence and presence of the cosolutes. Figure 3A shows UV melting curves of 2.0 µM oligonucleotides traced at 260 nm in 100 mM KCl buffer under dilute conditions. There was no hysteresis between the melting and annealing curves under all experimental conditions (Supplementary Figures S1 and S2), suggesting a two-state transition of the oligonucleotides between the random coil and the B-form duplex. The melting temperatures (*T*_m_) were evaluated and are listed in Table 1. The *T*_m_ value increased from 51.4 °C to 59.5 °C as the number of methylated cytosines increased from zero to eight. Previously, it was reported that methylation of cytosine increases the *T*_m_ value of a B-form duplex [30]. The increment of around 1 °C per one methylation observed here is consistent with previous reports [54]. Figure 3B–E show UV melting curves of 2.0 µM oligonucleotides in the presence of 30 wt% PEG200, TMAO, urea, and L-proline, respectively. The *T*_m_ values are listed in Table 1. As well as in the absence of the cosolutes, a stabilizing trend by methylation was observed. These results qualitatively show that methylation stabilizes the DNA duplexes regardless of dilute or molecular crowding conditions.

The *T*_m_ values of Me0 in the presence of PEG200, urea, and L-proline were decreased to 42.8, 37.5, and 40.0 °C, respectively, from that in the absence of cosolutes (51.4 °C). These results show that molecular crowding with the cosolutes reduces thermal stability of unmodified DNA duplexes. Conversely, the *T*_m_ of Me0 in the presence of 30 wt% TMAO (54.0 °C) increased. This stabilization by TMAO is consistent with a previous report [38]. It was observed that osmolytes having the trimethylamine group, such as TMAO, stabilize the DNA duplex at the lower concentrations because of a direct binding to a groove of the duplex through the formation of hydrogen bonds with nucleobases [38]. The *T*_m_ values of all oligonucleotides in the presence of 0, 10, 30, and 40 wt% of cosolutes are listed in Supplementary Table S1. The dependency of thermal stability (the *T*_m_ values) of the duplexes in the presence of various concentrations of the cosolutes will be discussed later. The *T*_m_ values of the methylated oligonucleotides (Me3, Me5, and Me8) also showed a similar trend: increase with TMAO and decrease with PEG200, urea, and L-proline. The degrees of favorable and unfavorable effects observed for the methylated oligonucleotides were almost identical with those of Me0. This suggests that the effects of cosolutes were similar for all oligonucleotides irrespective of methylation. This will be discussed in detail in the next section with the thermodynamic parameters.

### 2.4. The Combined Effects of Methylation and Cosolutes on the Thermodynamics of DNA Duplexes

**Figure 4:**
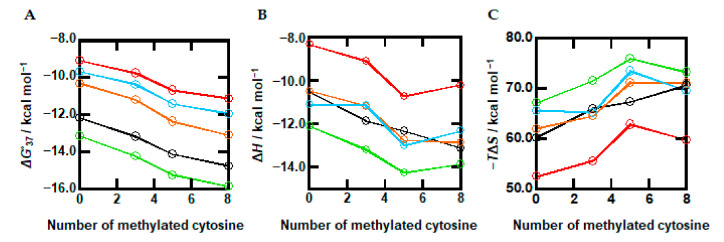
Plots of Δ*G*°_37_ (**A**), Δ*H*° (**B**), and −*T*Δ*S*° (**C**) as a function of methylated cytosine content for oligonucleotides. The parameters were evaluated in 100 mM KCl buffer in the absence of cosolutes (black) or in the presence of 30 wt% PEG200 (orange), TMAO (green), urea (red), or L-proline (blue). Standard deviation ≤ 0.3 (**A**), 3.7 (**B**), and 3.6 (**C**).

Based on the UV melting curves (Figure 3), we further calculated the thermodynamic parameters for B-form duplex formation of the oligonucleotides. Figure 4A shows plots of Δ*G*°_37_ vs. the number of methylations in the absence and presence of the cosolutes. The values of Δ*G*°_37_ in the absence of the cosolutes (black line) systematically decreased from −12.1 (Me0) to −13.1 (Me3), −14.1 (Me5), and −13.6 (Me8) kcal/mol, demonstrating quantitatively that methylation stabilized the duplex. We further observed that the Δ*G*°_37_ values of oligonucleotides in the presence of PEG200 decreased as the number of methylated cytosines increased, from −10.3 (Me0) to −11.2 (Me3), −12.3 (Me5), and −13.1 (Me8) kcal/mol. This stabilization effect by methylated cytosines was observed with the other cosolutes in a similar manner. Thus, the methylation effects on the thermodynamics of the DNA duplex are conserved and do not depend on the experimental conditions. The stabilization effect of methylation and its independence are consistent with previous studies for DNA i-motifs and triplex structures [32,39].

Figure 4B shows Δ*H*° vs. the number of methylated cytosines in the absence and presence of the cosolutes. The values of Δ*H*° in the absence of cosolutes (black line) gradually decreased as methylation decreased, from −72.5 (Me0) to −79.3 (Me3), −81.6 (Me5), and −85.5 (Me8) kcal/mol. This relationship shows that methylation stabilizes the DNA duplex in an enthalpic manner. The same trends were observed in the presence of the cosolutes, although the relationships included some fluctuations. Figure 4C shows plots of −*T*Δ*S*° in the absence and presence of cosolutes. Regardless of the presence of cosolutes, an increasing trend with more methylation was observed. These results suggest that the stabilization mechanism by methylation under molecular crowding conditions is the same as in a dilute solution. Therefore, we conclude that the methylation effect and the molecular crowding effect are independent and additive, in which the degree of the stabilization is the sum of the methylation and molecular crowding effects. Since the effects of methylation and molecular crowding are independent from each other, the combined effects are predictable from the individual effects. These results are useful to predict the properties of methylated DNA duplexes under cellular conditions and of PCR amplification in a test tube under various experimental conditions.

### 2.5. The Effect of Methylation on Hydration

**Figure 5:**
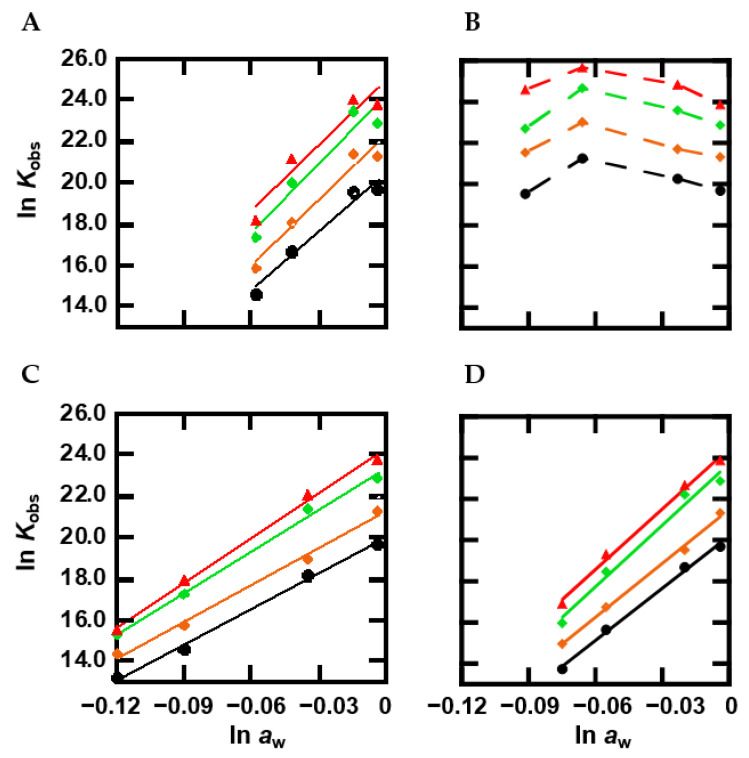
Plots of ln *K*_obs_ vs. ln *a*_w_ for DNA duplexes. Plots for Me0 (black circles), Me3 (orange diamonds), Me5 (green diamonds), and Me8 (red triangles) in KCl buffer with PEG200 (**A**), TMAO (**B**), urea (**C**) or L-proline (**D**).

Since it is generally considered that hydration of the DNA structure is an important factor determining the molecular crowding effect on structural stability [35], we further evaluated the hydration changes upon methylated duplex formation by observing the relationship between the equilibrium constant (*K*_obs_) and water activity (*a*_w_) as evaluated from osmotic pressure measurements [36,55]. Figure 5A shows plots of ln*K*_obs_ for the oligonucleotides vs. ln*a*_w_ in the presence of various concentration of the cosolutes. In the presence of PEG200, the plot showed a linear relationship. From the slope of the linear relationship, it is possible to evaluate the value of Δ*n*_w_, which is the number of water molecules taken up upon formation of a structure [56,57]. The values of Δ*n*_w_ were estimated to be 96, 105, 108, and 106 for Me0, Me3, Me5, and Me8, respectively, corresponding to an uptake of 9.6, 10.5, 10.8, and 10.6 water molecules per base pair, respectively. In the case of TMAO, the plot showed a convex shape irrespective of methylation. These nonlinear plots indicate that not only hydration, but also other factors are important for the effect of TMAO on duplex thermodynamic stability. Figure 5C shows the plots in the presence of various concentrations of urea. The plot increased linearly with an increase in ln*a*_w_. These slopes were estimated to be 57, 60, 67, and 72 for the oligonucleotides, which correspond to the uptake of 5.7, 6.0, 6.7, and 7.2, respectively, water molecules per base pair. L-proline also showed a linear relationship, and these slopes were 85, 87, 100, and 98 (8.5, 8.7, 10.0, and 9.8 water per base pair). Although the number of water molecules taken up upon duplex formation depended on the cosolute as shown in previous studies [42], a larger number was observed for more methylated cytosines. These results show that methylation enhances the number of water molecules that are taken up by oligonucleotides upon duplex formation.

## 3. Discussion

### 3.1. Structural Flexibility of Methylated DNA Duplexes under Molecular Crowding Conditions

Spectroscopic studies have shown that the overall structure of the DNA duplex is not largely affected by cytosine methylation [26,56,57,58]. However, it has been reported using single-molecule force spectroscopy that cytosine methylation has a significant effect on the separation of a duplex [59]. A single-molecule analysis by cyclization assay has further shown a reduction in DNA flexibility by methylation [60,61]. The decrement of DNA backbone flexibility has been observed in studies using nuclear magnetic resonance [24,31], and DNA transport using a nanopore [62]. Molecular dynamic simulations have further suggested that steric hindrance and hydrophobic effects of methyl groups reduce flexibility [63,64,65]. It has also been reported that the mutual relationship between stacking and hydrogen bonding interactions is affected by methylation, resulting in local distortions of DNA [66]. In this study, we observed a minor change in CD spectra depending on methylation as shown in Figure 2, in which the CD intensities of the positive and the negative peaks around 280 nm and 250 nm, respectively, varied depending on methylation. Such minor changes in CD spectra of methylated DNAs have also been observed in other studies [32,54,65]. Thus, the small changes observed here indicate that the local structure is affected by methylation. Moreover, the reduction of backbone flexibility of the methylated DNAs is consistent with the thermodynamic parameters shown in Figure 4. Comparing the values of Δ*G*°_37_ (Figure 4A), Δ*H*° (Figure 4B), and −*T*Δ*S*° (Figure 4C), thermodynamic stabilization with a larger number of methylated cytosines is accompanied with a more favorable enthalpy change that overcomes the unfavorable entropy change. In addition, the larger changes in the values of Δ*H*° and −*T*Δ*S*° were observed for Me5, indicating a larger local structure change of Me5. These results are consistent with the CD spectra shown in Figure 2, although these changes observed in Me5 are not sufficient to discuss quantitatively the specific local structure of Me5. These thermodynamic parameters clearly show the reduction of flexibility by methylation. Similar to the CD intensity change under dilute conditions, small CD intensity changes and the unfavorable enthalpy changes depending on methylation were observed under molecular crowding conditions (Figure 2 and Figure 4). These changes do not depend on the cosolute species. These results suggest that the effect of methylation on structural flexibility under molecular crowding conditions with the cosolutes is similar to that under dilute conditions.

### 3.2. Thermodynamics of DNA Duplexes with Methylations under Molecular Crowding Conditions

The *T*_m_ values (Table 1) and thermodynamic parameters (Figure 4) show that DNA duplexes are stabilized by methylation under both dilute and molecular crowding conditions. The plots of *T*_m_ and Δ*G*° vs. the number of methylations (Figure S3 and Figure 4A) show the slopes, which correspond to the average value of the *T*_m_ increment (Δ*T*_m_) and of the Δ*G*°_37_ decrement (ΔΔ*G*°_37_) per one methylation, which were 1.1 °C and −0.3 kcal/mol, respectively, under the dilute conditions. These values are consistent with previous reports showing that the Δ*T*_m_ and of the ΔΔ*G*°_37_ per one methylation are 0.5 ~1.5 °C [54,67] and around −0.9 kcal/mol [32]. In addition, the linear relationship between *T*_m_ and Δ*G*° and the number of methylations suggest that thermal and thermodynamic stability from methylations are additive and mostly independent of sequence context [32,68,69].

The values of Δ*T*_m_ per methylation under molecular crowding conditions with PEG200, urea, and L-proline were 1.3, 1.1, 1.2, and 1.1 °C, respectively. The ΔΔ*G*° per methylation under molecular crowding conditions with PEG200, TMAO, urea, and L-proline, were −0.4, −0.3, −0.3, and −0.3 kcal/mol, respectively. These values (Δ*T*_m_ and ΔΔ*G*°) are almost the same as those under the dilute conditions, and are mostly independent of cosolute species, showing that the methylation effect is conserved under molecular crowding conditions. The conservation of the methylation effect under molecular crowding has also been qualitatively reported by Shao’s group [39,70]. They studied methylation and hydroxymethylation effects on DNA duplex and i-motif structures and found that Δ*T*_m_ values of each after the introduction of methyl groups in the absence and presence of Ficoll are very similar with each other. These thermal analyses as well as our thermodynamic parameters prove that molecular crowding does not significantly alter the methylation regulation of DNA structures. The values of Δ*H*° (Figure 4B) and −*T*Δ*S*° (Figure 4C) showed that the enthalpic stabilization of the DNA duplex by methylation is also observed under the molecular crowding conditions, although the trend had some fluctuations. These parameters further confirm that the stabilization mechanism of the DNA duplex by methylation under molecular crowding conditions is the same as that under dilute conditions. Therefore, we conclude that the methylation effect and the molecular crowding effect on the DNA structures and their thermodynamics are independent and additive. The degree of the stabilization of the DNA duplex is the sum of the methylation and molecular crowding effects.

### 3.3. Stabilization Mechanism of DNA Duplexes by Methylation

Previous studies have proposed two possible mechanisms for how methylation affects the thermal and thermodynamic stability of DNA structures. One is that the hydrophobic effects of methyl groups affect the hydration state of DNA structures. A larger hydrophobicity by methylation has also been proposed to be important for nucleosome formation with histone proteins and the regulation of protein recognition. The other proposed mechanism is direct stabilization by introducing a stronger or an additional interaction by methylation. It has been reported that methylation facilitates stacking interactions [68,69,71], because the electron donating methyl group increases polarization of a cytosine base, leading to a higher dispersion energy [72]. In addition, a methyl group incorporates an enthalpically favorable interaction with neighboring bases [68,69,71,73].

To take a closer look at the methylation effects, we further evaluated the hydration state of the DNA duplexes with and without methylation. It has been reported that a DNA duplex takes up more water molecules upon folding [36]. Thus, duplex formation, which is a hydration reaction, should be interrupted under molecular crowding conditions where the concentration and activity of water molecules are reduced by the large amounts of cosolutes. In fact, we showed that molecular crowding destabilizes the canonical DNA duplex in an enthalpic manner, whereas molecular crowding stabilizes noncanonical DNA structures such as G-quadruplexes and triplexes in an enthalpic manner, because the noncanonical DNA structures release water molecules upon folding, leading to dehydration [36,74,75,76,77]. The number of water molecules taken up upon folding (Δ*n*_w_) can be calculated from the relationship between ln*K*_obs_, which is the equilibrium constant of structure formation, and ln*a*_w_, which is water activity [77]. As shown in Figure 5, a linear relationship in ln*K*_obs_ vs. ln*a*_w_ was observed for PEG200, urea, and L-proline, allowing us to evaluate the slope, which corresponds to the value of −Δ*n*_w_. Note that the nonlinear relationship for TMAO is consistent with previous studies [38] in which a direct interaction of TMAO with DNA was proposed for the convex upward relationship. It was observed that osmolytes having the trimethylamine group, such as TMAO, stabilize the DNA duplex at the lower concentrations because of a direct binding to a groove of the duplex through the formation of hydrogen bonds with nucleobases [38]. On the other hand, at the higher concentration, TMAO inhibits hydration reaction because of the preferential interaction with water molecules, leading to the destabilization of the duplex as shown in the other cosolutes. The slopes for PEG200, urea, and L-proline were positive, demonstrating hydration through folding. The values of −Δ*n*_w_ of Me0 with PEG200, urea, and L-proline were evaluated to be 96, 57, and 85, respectively (Table 2), indicating that these numbers of water molecules are taken up upon duplex formation. The value of Δ*n*_w_ is altered depending on cosolute species, which is consistent with our and other studies [36,37,74,75,76,77]. This is at least due to weak preferential interactions between cosolutes and water molecules. By comparing the differences in Δ*n*_w_ values with different cosolutes, methylation had a small effect. For example, Δ*n*_w_ slightly increased from 96 to 106 by eight methyl cytosines. Although a slight increment was also observed for urea and L-proline, again, the dependency on methylation was clearly smaller than that on the cosolute. These results suggest that methylation does not have a large effect on the number of water molecules taken up upon folding or the hydration state of the DNA duplex. This is consistent with a previous study showing that methylation does not affect the immobilization of water molecules in a DNA duplex or a DNA triplex [32]. Therefore, the enthalpic contribution of methylation to stabilize the DNA duplex does not originate from hydrophobic interactions of methyl groups. At least a part of the enthalpic contribution could be due to another factor arising from methylation.

What caused the enthalpic stabilization by methylation? As discussed above concerning the minor change in CD spectra with methylation, the methyl group can induce an additional interaction, leading to a more favorable enthalpy change that exceeds the unfavorable entropy change. Previously, to understand the stabilization effect of thymine over uracil, Cooper et al., carried out ab initio calculations for stacking interactions between base pairs [73]. They found that methyl group-neighboring nucleobase and methyl group-methyl group interactions stabilize DNA over its uracil counterpart, which lacks the methyl group, by up to 15% of the total stacking energy, corresponding to 2 kcal/mol. Under dilute conditions, the Δ*H*° value of Me0 was −72.5 kcal/mol and gradually decreased to be −85.5 kcal/mol for Me8 (Figure 4B). The slope, corresponding to ΔΔ*H*° per one methylation, was evaluated to be −1.6 kcal/mol. The values in the presence of PEG200, urea, and L-proline were −1.5, −1.2, and −0.8 kcal/mol, respectively. These experimental values obtained here are qualitatively consistent with the ab initio calculation. The ab initio calculation further suggested that methyl interactions with the adjacent base affect the twist angle and base pair distance (rise) of DNA, which may affect the −*T*Δ*S*° value. In fact, the −*T*Δ*S*° values of Me0 and Me8 were 60.3 kcal/mol and 70.8 kcal/mol (Figure 4C), resulting in a 1.3 kcal/mol unfavorable enthalpic contribution by one methyl group. An unfavorable entropic contribution is also observed under molecular crowding conditions with PEG200, urea, and L-proline. Therefore, the thermodynamic parameters indicate that a direct interaction and a local conformational change arising from methylation tune the thermodynamics of DNA duplexes under dilute and molecular crowding conditions.

### 3.4. Sequence Dependency of Methylation

**Figure 6:**
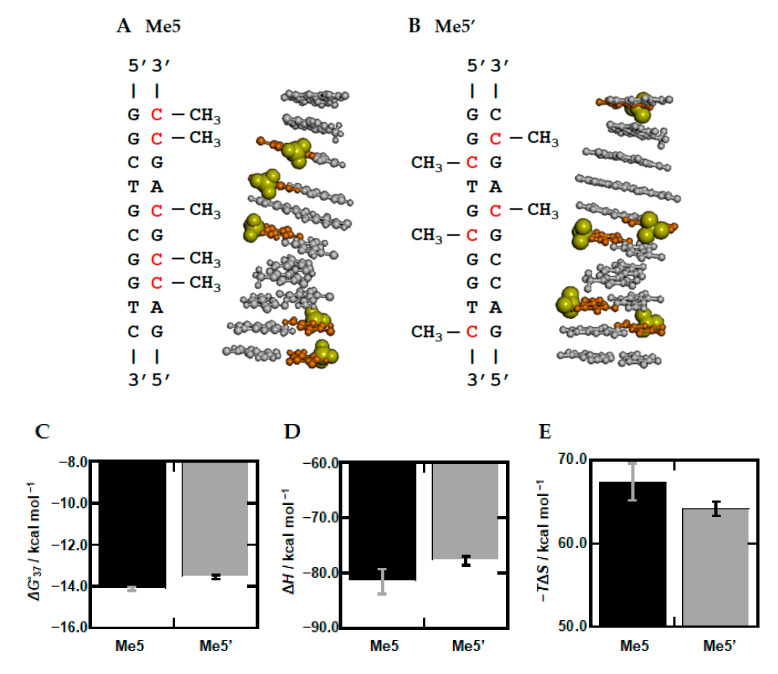
Sequences and schematic base position of Me5 and Me5′, and their thermodynamic parameters. Sequences and schematic base position of Me5 (**A**) and Me5′ (**B**). The backbone is omitted for clarify. Methylated cytosine bases and methyl groups are shown in red and as yellow VDW models, respectively. The values of Δ*G*°*_3_*_7_ (**C**), Δ*H*° (**D**), and −*T*Δ*S*° (**E**) are shown for Me5 (black) and Me5′ (gray) in 100 mM KCl buffer.

The above-mentioned ab initio calculation study further showed that two continuous thymine bases on the same strand have an additional stabilization of ~1 kcal/mol in comparison to two continuous methyl-deficient uracil bases due to an interaction between the contiguous methyl groups [73]. These results indicate that two continuous methylations on the same strand are energetically more favorable. Based on this, we further studied the nucleotide sequence effect of methylation. We designed Me5′, which has five discontinuous methylation sites, whereas Me5 includes two continuous methylation sites on the same strand (See sequence and position of the methylated cytosine bases in Me5 and Me5′, Figure 6A,B). Figure 6C–E show values of Δ*G*°, Δ*H*°, and −*T*Δ*S*° of Me5′, as well as Me5, respectively. We found that Me5′ is unstable by 0.56 kcal/mol. This destabilization is due to the unfavorable Δ*H*°, which exceeds the favorable −*T*Δ*S*°, demonstrating that the DNA duplex of Me5 which has two continuous methylation sites on the same strand is more stable than that of Me5′ with its enthalpic contribution. Therefore, these thermodynamic parameters are consistent with the methyl-methyl interactions proposed by the ab initio calculation study. In addition, Δ*n*_w_ values of Me5′ with PEG200, urea, and L-proline were 103, 58, and 92, respectively (Supplementary Figure S3 and Table S2). The difference between Δ*n*_w_ values of Me5′ and Me5 are within 10, supporting the direct methyl-methyl interaction, and indicating that the hydration state is not a major determinant of the thermodynamic properties of the DNA duplexes. From these results, we conclude that the continuous methylation sites induce a more stable and rigid structure, although more systematic DNA sequences with various methylation patterns and different nearest-neighbor sets should be studied to reveal rules for the sequence-dependent methylation effect. In addition, methylation is generally symmetrical at both strands. Although the oligonucleotides used in this study were not designed in consideration of this point, Me5′ involving two symmetric CpG sites showed the smaller effect on the local structure and its thermodynamics. These results imply that asymmetric methylation patterns lead to more significant changes in structure and stability of the DNA duplexes. Further studies considering the symmetric factor are required to reveal structure and stability of methylated CpG and non-CpG DNA duplexes existing in living cells.

## 4. Materials and Methods

### 4.1. Materials

All high-pressure liquid chromatography (HPLC)-grade DNA strands used in this study were acquired from Sigma-Aldrich Japan K. K. (Tokyo, Japan) and Hokkaido System Science Co., Ltd. (Hokkaido, Japan). Each oligonucleotide concentration was determined by the absorbance at 260 nm. Single-strand extinction coefficients were calculated from the mono- and dinucleotide data using the nearest-neighbor approximation model. The absorbance was measured using a UV-1800 spectrophotometer (Shimadzu, Kyoto, Japan). Chemical reagents were purchased from Wako Pure Chemical Co., Ltd. (Osaka, Japan) or Tokyo Chemical Industry Co., Ltd. (Tokyo, Japan).

### 4.2. CD Spectroscopy

Circular dichroism (CD) spectra of oligonucleotides were measured for 20 µM oligonucleotides in buffer containing 100 mM KCl, 10 mM K_2_HPO_4_ (pH 7.0), and 1 mM K_2_EDTA in the absence or presence of 40 wt% cosolute at 25 °C using a Jasco J-820 spectropolarimeter (JASCO Co., Ltd., Tokyo, Japan) with a quartz cell with 0.1 cm path length. Before measurement, each sample was heated to 90 °C for 5 min and gently cooled to 0 °C at a rate of 0.5 °C/min.

### 4.3. Thermal and Thermodynamic Analysis

The melting curves of each oligonucleotide were measured by monitoring the absorption at 260 nm using a UV-1800 spectrophotometer (Shimadzu Co., Ltd., Kyoto, Japan) with a temperature controller. DNA samples in buffer containing 100 mM KCl, 10 mM K_2_HPO_4_ (pH 7.0), and 1 mM K_2_EDTA in the absence or presence of various concentrations of cosolutes were heated at a rate of 0.5 °C/min from 0 to 93 °C to trace the thermal denaturation curves. The thermodynamic parameters were calculated from the melting curves by a curve fitting procedure as described previously [78,79].

### 4.4. Water Activity Measurements

The activity of water molecules was determined by the osmotic stressing method through vapor phase osmometry using a pressure osmometer (5520XR, Wescor, Logan, UT, USA) at 25 °C [36,38].

## 5. Conclusions

In this study, we examined the thermodynamics of DNA oligonucleotides with various numbers of methylation under dilute and cell-mimicking molecular crowding conditions with different cosolutes. The thermodynamic parameters as well as CD spectra demonstrated that the effects of methylation and molecular crowding are independent and additive. Of note, the thermodynamic parameters had a linear relationship with the number of methylations and the degree of molecular crowding. These results may be useful to predict the properties of DNA duplexes with methylation in cell-mimicking conditions. Moreover, we found that the stabilization effect of methylation can be ascribed to the favorable enthalpic contribution, suggesting that direct interactions of the methyl groups with adjacent bases and adjacent methyl groups play a role in determining the flexibility and thermodynamics of DNA duplexes. Finally, although the difference between Me5 and Me5′ observed here is small, this difference indicates that methylation pattern and position alter the flexibility and thermodynamics of DNA duplexes, and hence may affect the binding of proteins such as histones and others, leading to epigenetic regulation of gene expression. Further studies with more systematic DNA sequences with different sets of neighboring base pairs may be required to predict how conformational flexibility and stability depend on nucleotide sequence and epigenetic modifications, which will in turn shed light on the roles of DNA flexibility and thermodynamics in the epigenetic regulation of gene expression.

## Figures and Tables

**Table 1:** Melting temperature (*T*_m_) ^1^ (°C) for 2.0 µM oligonucleotides in the absence or presence of 30 wt% cosolutes (PEG200, TMAO, urea, or L-proline) ^2^.

Abbreviation	Cosolutes				
	Without Cosolute	PEG200	TMAO	Urea	L-Proline
Me0	51.4 ± 0.5^2^	42.8 ± 0.0	54.0 ± 0.2	37.5 ± 0.0	40.0 ± 0.1
Me3	54.4 ± 0.5	46.4 ± 0.2	57.2 ± 0.1	40.7 ± 0.1	42.8 ± 0.2
Me5	58.0 ± 0.5	50.1 ± 0.2	59.9 ± 0.3	44.4 ± 0.2	46.2 ± 0.4
Me8	59.5 ± 1.8	52.9 ± 0.2	63.0 ± 0.3	46.7 ± 0.2	48.7 ± 0.1

^1^ Melting temperature was measured at 2.0 μM strand concentration. ^2^ Values are meant ± standard deviation from at least three measurements.

**Table 2:** The value of −Δ*n*_w_ for Me0, Me3, Me5, and Me8 in the buffer containing 100 mM KCl, 10 mM K_2_HPO_4_, and 1 mM K_2_EDTA with PEG200, TMAO, urea, or L-proline ^1^.

Abbreviation	−Δ*n*_w_			
	PEG200	TMAO ^2^	Urea	L-Proline
Me0	96 ± 4	-	57 ± 1	85 ± 3
Me3	105 ± 2	-	60 ± 2	87 ± 1
Me5	108 ± 2	-	67 ± 1	100 ± 3
Me8	106 ± 11	-	72 ± 3	98 ± 8

^1^ Values are meant ± standard deviation from at least three individual plots of ln *K*_obs_ vs. ln *a*_w_. ^2^ −Δ*n*_w_ with TMAO was not possible to evaluate, because of the non-linear relationship.

## Data Availability

Not applicable.

## Supplementary Materials

The following are available online at https://www.mdpi.com/1422-0067/22/2/947/s1, Figure S1: Melting and annealing curves of 2.0 µM Me0, Figure S2: Melting and annealing curves of 2.0 µM Me8, Figure S3: Plots of Tm values vs. number of methylated cytosines for Me0, Me3, Me5, and Me8, Table S1: The Tm values of all oligonucleotides in the presence of 0, 10, 30, and 40 wt% of cosolutes, Table S2: The values of −Δnw for Me5 and Me5′.

## References

[1] Holliday R., Pugh J.E. (1975). DNA modification mechanisms and gene activity during development. Science.

[2] Smith Z.D., Meissner A. (2013). DNA methylation: Roles in mammalian development. Nat. Rev..

[3] Song J., Rechkoblit O., Bestor T.H., Patel D.J. (2011). Structure of DNMT1-DNA complex reveals a role for autoinhibition in maintenance DNA methylation. Science.

[4] Ehrich M., Gama-Sosa M.A., Huang L.-H., Midgett R.M., Kuo K.C., Mccune R.A., Gehrke C. (1982). Amount and distribution of 5-methylcytosine in human DNA from different types of tissues or cells. Nucleic Acid Res..

[5] Bird A.P., Taggart M.H., Nicholls R.D., Higgs D.R. (1987). Non-methylated CpG-rich islands at the human α-globin locus: Implications for evolution of the α-globin pseudogene. EMBO J..

[6] Bird A.P. (2002). DNA methylation patterns and epigenetic memory. Genes Dev..

[7] Cokus S.J., Feng S., Zhang X., Chen Z., Merriman B., Haudenschild C.D., Pradhan S., Nelson S.F., Pellegrini M., Jacobsen S.E. (2008). Shotgun bisulphite sequencing of the Arabidopsis genome reveals DNA methylation patterning. Nature.

[8] Lister R., O’Malley R.C., Tonti-Filippini J., Gregory B.D., Berry C.C., Millar A.H., Ecker J.R. (2008). Highly integrated single-base resolution maps of the epigenome in Arabidopsis. Cell.

[9] Lister R., Pelizzola M., Dowen R.H., Hawkins R.D., Hon G., Tonti-Filippini J., Joseph R.N., Lee L., Ye Z., Ngo Q.-M. (2009). Human DNA methylomes at base resolution show widespread epigenomic differences. Nature.

[10] Shirane K., Toh H., Kobayashi H., Kono T., Sasaki H. (2013). Mouse oocyte methylomes at base resolution reveal genome-wide accumulation of non-CpG methylation and role of DNA methyltransferases. PLoS Genet..

[11] Laurent L., Wong E., Li G., Huynh T., Tsirigos A. (2010). Dynamic changes in the human methylome during differentiation. Genome Res..

[12] Ramsahoye B.H., Biniszkiewicz D., Lyko F., Clark V., Bird A.P., Jaenisch R. (2000). Non-CpG methylation is prevalent in embryonic stem cells and may be mediated by DNA methyltransferase 3a. Proc. Natl. Acad. Sci. USA.

[13] Haines T.R., Rodenhiser D.I., Ainsworth P.J. (2001). Allele-specific non-CpG methylation of the Nf1 gene during early mouse development. Dev. Biol..

[14] Lister R., Pelizzola M., Kida Y.S., Hawkins R.D., Nery J.R., Hon G., Antosiewicz-Bourget J., O’Mally R., Castanon R., Klugman S. (2011). Hotspots of aberrant epigenomic reprogramming in human induced pluripotent stem cells. Nature.

[15] Jaenisch R., Bird A. (2003). Epigenetic regulation of gene expression: How the genome integrates intrinsic and environmental signals. Nat. Genet..

[16] Riggs A.D. (1975). X inactivation, differentiation, and DNA methylation. Cytogenet. Cell Genet..

[17] Jones P.A. (2012). Functions of DNA methylation: Islands, start sites, gene bodies and beyond. Nat. Rev..

[18] Robertson K.D., Jones P.A. (2000). DNA methylation: Past, present and future directions. Carcinogenesis.

[19] Dawson M.A., Kouzarides T. (2012). Cancer Epigenetics: From Mechanism to Therapy. Cell.

[20] Hwang J.-Y., Aromolaran K.A., Zukin R.S. (2017). The Emerging Field of Epigenetics in Neurodegeneration and Neuroprotection. Nat. Rev. Neurosci..

[21] Buck-Koehntop B.A., Defossez P.-A. (2013). On how mammalian transcription factors recognize methylated DNA. Epigenetics.

[22] Schübeler D. (2015). Function and information content of DNA methylation. Nature.

[23] Luo C., Ecker J.R. (2015). Exceptional epigenetics in the brain. Science.

[24] Geahigan K.B., Meints G.A., Hatcher M.E., Orban J., Drobny G.P. (2000). The Dynamic impact of CpG methylation in DNA. Biochemistry.

[25] Lefebvre A., Mauffret O., Antri S.E., Monnot M., Lescot E., Fermandlian S. (1995). Sequence dependent effects of CpG methylated cytosine A joint ^1^H-NMR and ^31^P-NMR study. Eur. J. Biochem..

[26] Renciuk D., Blacpue O., Vorlickova M., Spingler B. (2013). Crystal structures of B-DNA dodecamer containing the epigenetic modifications 5-hydroxymethylcytosine or 5-methylcytosine. Nucleic Acids Res..

[27] Mayer-Jung C., Moras D., Timsit Y. (1997). Effect of methylation on DNA-DNA recognition at CpG steps. J. Mol. Biol..

[28] Hodges-Garcia Y., Hagerman P.J. (1992). Methylated cytosine can induce local distortions in the structure of duplex DNA. Biochemistry.

[29] Nardo L., Lamperti M., Salerno D., Cassina V., Missana N., Bondani M., Tempestini A., Mantegazza F. (2015). Effects of non-CpG site methylation on DNA thermal stability: A fluorescence study. Nucleic Acids Res..

[30] Mayer-Jung C., Moras D., Timsit Y. (1998). Hydration and recognition of methylated CpG steps in DNA. EMBO J..

[31] Derreumaux S., Chaoui M., Tevanian G., Fermandjian S. (2001). Impact of CpG methylation on structure, dynamics and solvation of cAMP DNA responsive element. Nucleic Acids Res..

[32] Carr C.E., Ganugula R., Shikiya R., Soto A.M., Marky L.A. (2018). Effects of dC-d(m^5^C) substitution on the folding of intramolecular triplexes with mixed TAT and C^+^GC base triplets. Biochemie.

[33] Wang S., Kool E.T. (1995). Origins of the large differences in stability of DNA and RNA helices: C-5 methyl and 2′-hydroxyl effects. Biochemistry.

[34] Zendlova L., Hobza P., Kabelac M. (2006). Potential energy surfaces of the microhydrated guanine-cytosine base pair and its methylated analogue. ChemPhysChem.

[35] Nakano S., Miyoshi D., Sugimoto N. (2014). Effects of molecular crowding on the structures, interactions, and functions of nucleic acids. Chem. Rev..

[36] Miyoshi D., Karimata H., Sugimoto N. (2006). Hydration regulates thermodynamics of G-quadruplex formation under molecular crowding conditions. J. Am. Chem. Soc..

[37] Miyoshi D., Sugimoto N. (2008). Molecular crowding effects on structure and stability of DNA. Biochimie.

[38] Ueda Y., Zouzumi Y., Maruyama A., Nakano S., Sugimoto N., Miyoshi D. (2016). Effects of trimethylamine N-oxide and urea on DNA duplex and G-quadruplex. Sci. Technol. Adv. Mater..

[39] Xu B., Devi G., Shao F. (2015). Regulation of telomeric i-motif stability by 5-methylcytosine and 5-hydroxymethylcytosine modification. Org. Biomol. Chem..

[40] Spink C.H., Chairs J.B. (1999). Effects of Hydration, Ion Release, and Excluded Volume on the Melting of Triplex and Duplex DNA. Biochemistry.

[41] Goobes R., Kahana N., Cohen O., Minsky A. (2003). Metabolic buffering exerted by macromolecular crowding on DNA-DNA interactions: Origin and physiological significance. Biochemistry.

[42] Ilia B., Bolen D.W. (1998). Forcing Thermodynamically Unfolded Proteins to Fold. J. Biol. Chem..

[43] Miyoshi D., Nakao A., Sugimoto N. (2002). Molecular Crowding Regulates the Structural Switch of the DNA G-Quadruplex. Biochemistry.

[44] Takahashi S., Yamamoto J., Kitamura A., Kinjo M., Sugimoto N. (2019). Characterization of intracellular crowding environment with topology-based DNA quadruplex sensors. Anal. Chem..

[45] Qu Y., Bolen C.L., Bolen D.W. (1998). Osmolyte- driven contraction of a random coil protein. Proc. Natl. Acad. Sci. USA.

[46] Lin T.Y., Timasheff S.N. (1994). Why Do Some Organisms Use a Urea-Methylamine Mixture as Osmolyte? Thermodynamic Compensation of Urea and Trimethylamine N-Oxide Interactions with Protein. Biochemistry.

[47] Bennion B.J., Daggett V. (2003). The molecular basis for the chemical denaturation of proteins by urea. Proc. Natl. Acad. Sci. USA.

[48] Canchi D.R., Paschek D., Garcia A.E. (2010). Equilibrium Study of Protein Denaturation by Urea. J. Am. Chem. Soc..

[49] Guinn E.J., Schwinefus J.J., Cha H.K., McDevitt J.L., Merker W.E., Ritzer R., Muth S.W., Engelsgjerd S.W., Mangold K.E., Thompson P.J. (2013). Quantifying Functional Group Interactions That Determine Urea Effects on Nucleic Acid Helix Formation. J. Am. Chem. Soc..

[50] Diehl R.C., Guinn E.J., Capp M.W., Tsodikov O.V., Record M.T. (2013). Quantifying additive interactions of the osmolyte proline with individual functional groups of proteins: Comparisons with urea and glycine betaine, interpretation of m-values. Biochemistry.

[51] Rajendrakumar C.S., Suryanarayana T., Reddy A.R. (1997). DNA helix destabilization by proline and betaine: Possible role in the salinity tolerance process. FEBS Lett..

[52] Auton M., Bolen D.W., Rösgen J. (2008). Structural thermodynamics of protein preferential solvation: Osmolyte solvation of proteins, aminoacids, and peptides. Proteins.

[53] Riazance J.H., Baase W.A., Johnson W.C., Hall K., Cruz P., Tinoco I. (1985). Evidence for Z-form RNA by vacuum UV circular dichroism. Nucleic. Acids Res..

[54] Thalhammer A., Hansen A.S., El-Sagheer A.H., Brownb T., Schofield C.J. (2011). Hydroxylation of methylated CpG dinucleotides reverses stabilisation of DNA duplexes by cytosine 5-methylation. Chem. Commun..

[55] Nakano S.-I., Karimata H., Ohmichi T., Kawakami J., Sugimoto N. (2004). The effect of molecular crowding with nucleotide length and cosolute structure on DNA duplex stability. J. Am. Chem. Soc..

[56] Theruvathu J.A., Yin Y.W., Pettitt B.M., Sowers L.C. (2013). Comparison of the structural and dynamic effects of 5-methylcytosine and 5-chlorocytosine in a CpG dinucleotide sequence. Biochemistry.

[57] Lercher L., McDonough M.A., El-Sagheer A.H., Thalhammer A., Kriaucionis S., Brown T., Schofield C.J. (2014). Structural insights into how 5-hydroxymethylation influences transcription factor binding. Chem. Commun..

[58] Szulik M.W., Pallan P.S., Nocek B., Voehler M., Banerjee S., Brooks S., Joachimiak A., Egli M., Eichman B.F., Stone M.P. (2015). Differential Stabilities and Sequence-Dependent Base Pair Opening Dynamics of Watson−Crick Base Pairs with 5-Hydroxymethylcytosine, 5-Formylcytosine, or 5-Carboxylcytosine. Biochemistry.

[59] Severin P.M.D., Zou X., Gaub H.E., Schulten K. (2011). Cytosine methylation alters DNA mechanical properties. Nucleic Acids Res..

[60] Ngo T.T.M., Yoo J., Dai Q., Zhang Q., He C., Aksimentiev A., Ha T. (2016). Effects of cytosine modifications on DNA flexibility and nucleosome mechanical stability. Nat. Commun..

[61] Nathan D., Crothers D.M. (2002). Bending and flexibility of methylated and unmethylated EcoRI DNA. J. Mol. Biol..

[62] Mirsaidov U., Timp W., Zou X., Dimitrov V., Schulten K., Feinberg A.P., Timp G. (2009). Nanoelectromechanics of methylated DNA in a synthetic nanopore. Biophys. J..

[63] Luger K., Mader A.W., Richmond R.K., Sargent D.F., Richmond T.J. (1997). Crystal structure of the nucleosome core particle at 2.8 angstrom resolution. Nature.

[64] Churchman L.S., Weissman J.S. (2011). Nascent transcript sequencing visualizes transcription at nucleotide resolution. Nature.

[65] Acosta-Silva C., Branchadell V., Bertran J., Oliva A. (2010). Mutual Relationship between Stacking and Hydrogen Bonding in DNA. Theoretical Study of Guanine-Cytosine, Guanine-5-methylcytosine, and Their Dimers. J. Phys. Chem. B.

[66] Hognon C., Besancenot V., Gruez A., Grandemange S., Monari A. (2019). Cooperative Effects of Cytosine Methylation on DNA Structure and Dynamics. J. Phys. Chem. B.

[67] Madugundu G.S., Cadet J., Wagner J.R. (2014). Hydroxyl-radical-induced oxidation of 5-methylcytosine in isolated and cellular DNA. Nucleic Acids Res..

[68] Plum G.E., Park Y.-W., Singleton S.F., Dervan P.B., Breslauer K.J. (1990). Thermodynamic characterization of the stability and the melting behavior of a DNA triplex: A spectroscopic and calorimetric study. Proc. Natl. Acad. Sci. USA.

[69] Roberts R.W., Crothers D.M. (1992). Stability and properties of double and triple helices: Dramatic effects of RNA or DNA backbone composition. Science.

[70] Wu Q., Wong J.R., Yeo P.L.Q., Zhang D., Shao F. (2016). Methylation on CpG repeats modulates hydroxymethylcytosine induced duplex destabilization. RSC Adv..

[71] Povsic T.J., Dervan P.B. (1989). Triple helix formation by oligonucleotides on DNA extended to the physiological pH range. J. Am. Chem. Soc..

[72] Tretyakova N., Guza R., Matter B. (2008). Endogenous cytosine methylation and the formation of carcinogen carcinogen–DNA adducts. Nucleic Acids Symp. Ser..

[73] Cooper V.R., Thonhauser T., Puzder A., Schroder E., Lundqvist B.I., Langreth D.C. (2008). Stacking Interactions and the Twist of DNA. J. Am. Chem. Soc..

[74] Yu H., Gu X., Nakano S., Miyoshi D., Sugimoto N. (2012). Beads-on-a-String Structure of Long Telomeric DNAs under Molecular Crowding Conditions. J. Am. Chem. Soc..

[75] Muhuri S., Mimura K., Miyoshi D., Sugimoto N. (2009). Stabilization of Three-Way Junctions of DNA under Molecular Crowding Conditions. J. Am. Chem. Soc..

[76] Miyoshi D., Nakamura K., Tateishi-Karimata H., Ohmichi T., Sugimoto N. (2009). Hydration of Watson-Crick Base Pairs and Dehydration of Hoogsteen Base Pairs Inducing Structural Polymorphism under Molecular Crowding Conditions. J. Am. Chem. Soc..

[77] Miyoshi D., Shizuka M., Nakano S., Sugimoto N. (2004). Duplex Dissociation of Telomere DNAs Induced by Molecular Crowding. J. Am. Chem. Soc..

[78] Sugimoto N., Nakano S., Katoh M. (1995). Thermodynamic parameters to predict stability of RNA/DNA hybrid duplexes. Biochemistry.

[79] Nakano S., Fujimoto M., Hara H. (1999). Nucleic acid duplex stability: Influence of base composition on cation effects. Nucleic Acids Res..

