# Supplementary material for: Combined Effects of Methylated Cytosine and Molecular Crowding on the Thermodynamic Stability of DNA Duplexes
Source: Int J Mol Sci. 2021 Jan 19;22(2):947. doi: 10.3390/ijms22020947 (PMC7833394; doi:10.3390/ijms22020947)
Supplement: Supplementary file 1 [file ijms-22-00947-s001.pdf]

Supporting information

Combined effects of methylated cytosine and molecular crowding on the thermodynamic stability of DNA duplexes

Mitsuki Tsuruta [1], Yui Sugitani [1], Naoki, Sugimoto [1,2] and Daisuke Miyoshi [1,*]

[1] Faculty of Frontiers of Innovative Research in Science and Technology (FIRST), Konan University, Kobe 650-0047, Japan.
[2] Frontier Institute for Biomolecular Engineering Research (FIBER), Konan University, Kobe 650-0047, Japan.
* Correspondence: miyoshi@konan-u.ac.jp; Tel.: +81-078-303-1426

Figure S1: UV-Melting Curves for 2.0 μM Me0 oligonucleotides in a buffer containing 100 mM KCl, 10 mM K_2HPO_4 (pH 7.0), and 1 mM K_2EDTA in the absence of cosolutes (a) or in the presence of 30 wt% PEG200 (b), TMAO (c), urea (d), or L-proline (e). UV melting curves were traced at 260 nm. Red and black lines show annealing and melting curve, respectively.

Figure S2: UV-Melting Curves for 2.0 μM Me8 oligonucleotides in a buffer containing 100 mM KCl, 10 mM K_2HPO_4 (pH 7.0), and 1 mM K_2EDTA in the absence of cosolutes (a) or in the presence of 30 wt% PEG200 (b), TMAO (c), urea (d), or L-proline (e). UV melting curves were traced at 260 nm. Red and black lines show annealing and melting curve, respectively.

Figure S3. Plots of T_m values versus number of methylated cytosine for Me0, Me3, Me5, and Me8 in a buffer containing 100 mM KCl, 10 mM K_2HPO_4 (pH 7.0), and 1 mM K_2EDTA in the absence of cosolutes (black) or in the presence of 30wt% PEG200 (orange), TMAO (green), urea (red), and L-proline (blue).

Table S1: The T_m values of all oligonucleotides in the presence of 0, 10, 30, 40 wt% of cosolutes (PEG200, TMAO, urea, and L-proline)

Table S2: The value of $-\Delta n_w$ for oligonucleotides (Me5 and 5′) in a buffer containing 100 mM KCl, 10 mM K_2HPO_4 (pH 7.0), and 1 mM K_2EDTA

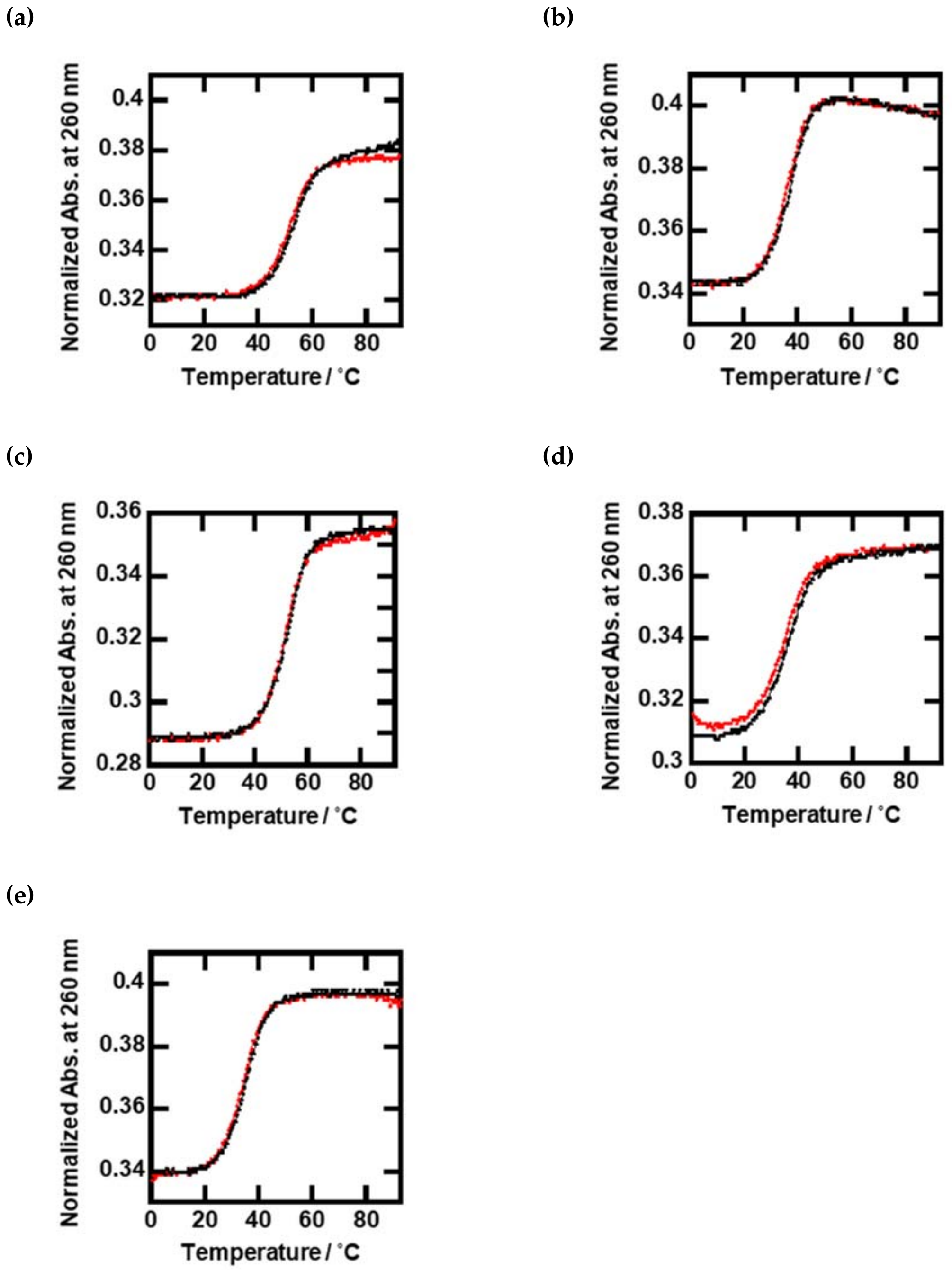

Figure S1: UV-Melting Curves for 2.0 μM Me0 oligonucleotides in a buffer containing 100 mM KCl, 10 mM K_2HPO_4 (pH 7.0), and 1 mM K_2EDTA in the absence of cosolutes (a) or in the presence of 30 wt% PEG200 (b), TMAO (c), urea (d), or L-proline (e). UV melting curves were traced at 260 nm. Red and black lines show annealing and melting curve, respectively.

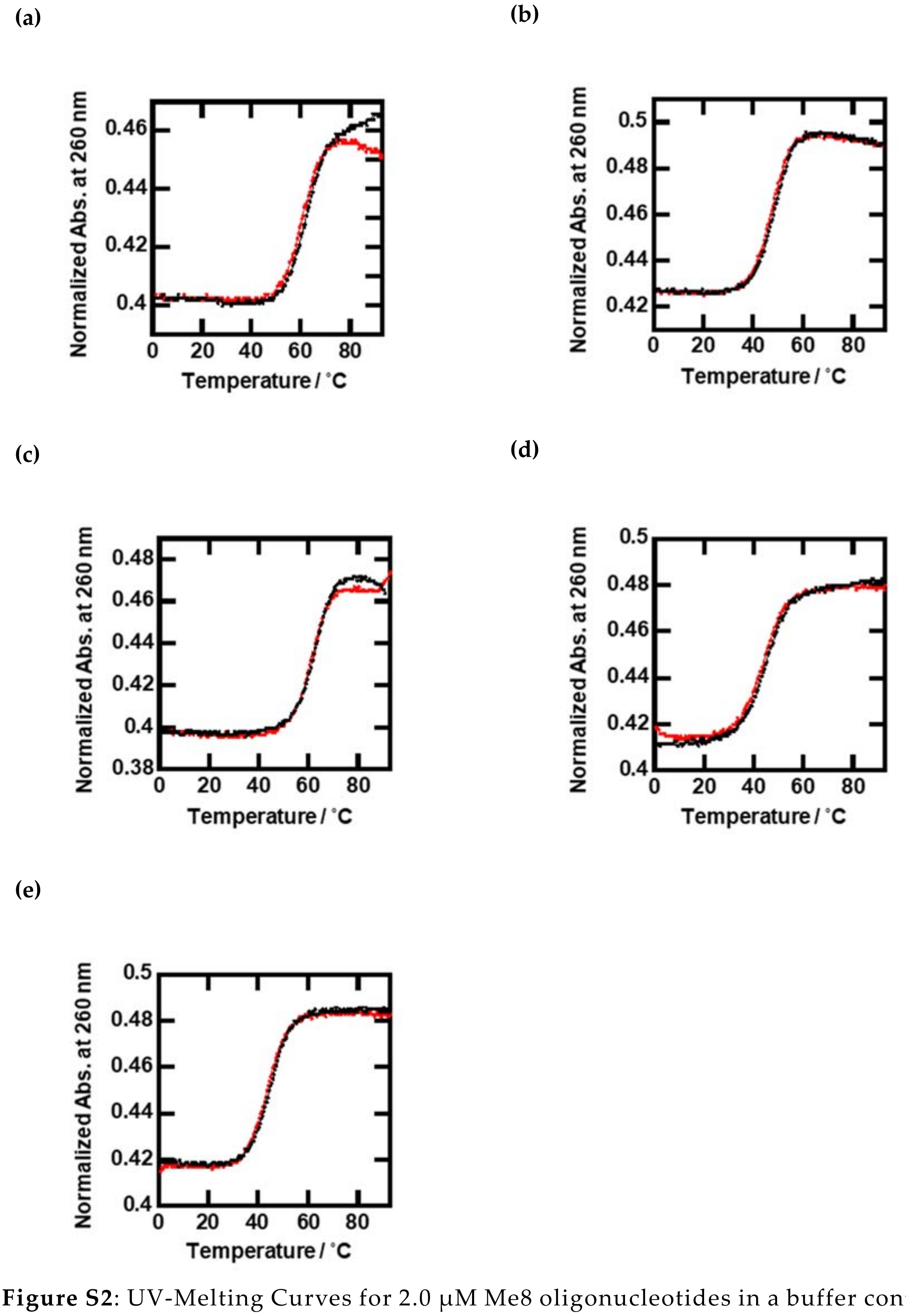

Figure S2: UV-Melting Curves for 2.0 μM Me8 oligonucleotides in a buffer containing 100 mM KCl, 10 mM K_2HPO_4 (pH 7.0), and 1 mM K_2EDTA in the absence of cosolutes (a) or in the presence of 30 wt% PEG200 (b), TMAO (c), urea (d), or L-proline (e). UV melting curves were traced at 260 nm. Red and black lines show annealing and melting curve, respectively.

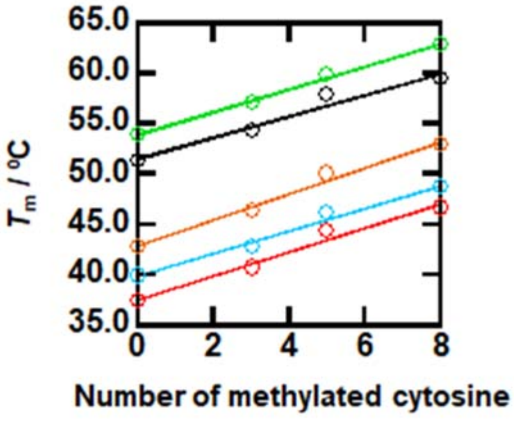

Figure S3: Plots of T_m values versus number of methylated cytosine for Me0, Me3, Me5, and Me8 in a buffer containing 100 mM KCl, 10 mM K_2HPO_4 (pH 7.0), and 1 mM K_2EDTA in the absence of cosolutes (black) or in the presence of 30wt% PEG200 (orange), TMAO (green), urea (red), and L-proline (blue).

Table S1: Melting temperature (T_m)[1] (°C) for 2.0 μM oligonucleotides in the various wt% of cosolutes (PEG200, TMAO, urea, or L-proline)[2]

Abbreviation	wt%	T_m (°C)			
		**PEG200**	**TMAO**	**urea**	**L-proline**
Me0	0	51.4 ± 0.5			
	10	49.8 ± 0.1	53.2 ± 0.3	47.3 ± 0.2	47.6 ± 0.5
	30	42.8 ± 0.1	54.0 ± 0.2	37.5 ± 0.1	40.0 ± 0.1
	40	37.4 ± 0.4	50.8 ± 1.6	33.2 ± 0.1	34.9 ± 0.1
Me3	0	54.4 ± 0.5			
	10	52.9 ± 0.3	56.4 ± 0.3	49.6 ± 0.2	50.3 ± 0.1
	30	46.4 ± 0.2	57.2 ± 0.1	40.7 ± 0.1	42.8 ± 0.2
	40	40.6 ± 0.1	55.1 ± 0.5	36.5 ± 0.4	38.2 ± 0.1
Me5	0	58.0 ± 0.5			
	10	56.7 ± 0.2	59.7 ± 0.3	53.9 ± 0.3	54.6 ± 0.1
	30	50.1 ± 0.2	59.9 ± 0.3	44.4 ± 0.2	46.2 ± 0.4
	40	44.3 ± 0.2	58.3 ± 0.7	39.4 ± 0.3	40.8 ± 0.4
Me8	0	59.5 ± 1.8			
	10	59.0 ± 0.2	62.4 ± 0.1	56.3 ± 0.1	56.9 ± 0.4
	30	52.9 ± 0.2	63.0 ± 0.3	46.7 ± 0.2	48.7 ± 0.1
	40	47.4 ± 0.4	62.3 ± 1.1	40.2 ± 1.3	43.2 ± 0.2

[1] Melting temperature was measured at 2.0 μM strand concentration.
[2] Values are meant ± standard deviation from at least three measurements.

Table S2: The value of $-\Delta n_w$ for oligonucleotides (Me5 and 5′) in a buffer containing 100 mM KCl, 10 mM K_2HPO_4 (pH 7.0), and 1 mM K_2EDTA

**Abbreviation**	$-\Delta n_w$			
	**PEG200**	**TMAO**	**urea**	**L-proline**
GC8Me5	108	-	67	100
GC8Me5′	103	-	58	92
